# Health effects of exposure to diesel exhaust in diesel-powered trains

**DOI:** 10.1186/s12989-019-0306-4

**Published:** 2019-06-11

**Authors:** Maria Helena Guerra Andersen, Marie Frederiksen, Anne Thoustrup Saber, Regitze Sølling Wils, Ana Sofia Fonseca, Ismo K. Koponen, Sandra Johannesson, Martin Roursgaard, Steffen Loft, Peter Møller, Ulla Vogel

**Affiliations:** 1 Department of Public Health, Section of Environmental Health, University of Copenhagen, Øster Farimagsgade 5A, DK-1014 Copenhagen K, Denmark; 2 The National Research Centre for the Working Environment, Lersø Parkalle 105, DK-2100 Copenhagen Ø, Denmark; 3 Department of Occupational and Environmental Medicine, Sahlgrenska Academy at University of Gothenburg, Gothenburg, Sweden; 4 DTU Health Tech., Technical University of Denmark, DK-2800 Kgs. Lyngby, Denmark

**Keywords:** Diesel exhaust, Train exposure, Lung function, Cardiovascular function, DNA damage, Comet assay

## Abstract

**Background:**

Short-term controlled exposure to diesel exhaust (DE) in chamber studies have shown mixed results on lung and systemic effects. There is a paucity of studies on well-characterized real-life DE exposure in humans. In the present study, 29 healthy volunteers were exposed to DE while sitting as passengers in diesel-powered trains. Exposure in electric trains was used as control scenario. Each train scenario consisted of three consecutive days (6 h/day) ending with biomarker samplings.

**Results:**

Combustion-derived air pollutants were considerably higher in the passenger carriages of diesel trains compared with electric trains. The concentrations of black carbon and ultrafine particles were 8.5 μg/m^3^ and 1.2–1.8 × 10^5^ particles/cm^3^ higher, respectively, in diesel as compared to electric trains. Net increases of NOx and NO_2_ concentrations were 317 μg/m^3^ and 36 μg/m^3^. Exposure to DE was associated with reduced lung function and increased levels of DNA strand breaks in peripheral blood mononuclear cells (PBMCs), whereas there were unaltered levels of oxidatively damaged DNA, soluble cell adhesion molecules, acute phase proteins in blood and urinary excretion of metabolites of polycyclic aromatic hydrocarbons. Also the microvascular function was unaltered. An increase in the low frequency of heart rate variability measures was observed, whereas time-domain measures were unaltered.

**Conclusion:**

Exposure to DE inside diesel-powered trains for 3 days was associated with reduced lung function and systemic effects in terms of altered heart rate variability and increased levels of DNA strand breaks in PBMCs compared with electric trains.

**Trial registration:**

ClinicalTrials.Gov (NCT03104387). Registered on March 23rd 2017

## Background

Diesel exhaust (DE) exposure occurs in both environmental and occupational settings where engines are used for transportation or heavy-duty equipment for work processes. Exhaust from on-road vehicles is generally the most important source of DE in the urban environment, but emissions from diesel trains can also be an important local source of DE. The diesel trains do not only affect the DE levels in outdoor air, but also inside the trains since the plume may penetrate the train interior, which is a special problem when the locomotive pulls the train [1, 2]. Accordingly, both the staff and commuters may be exposed to DE on a daily basis. Long-term exposure to DE is associated with increased risk of lung cancer [3, 4]. In addition, traffic-generated air pollution is considered to be an important risk factor for cardiovascular diseases [5]. Although ultrafine particles (UFP) from diesel-powered vehicles are considered to be an important contributor to cardiovascular disease, it is difficult to separate this effect from other sources of particulate matter (PM) in urban air as diesel vehicles only contribute to 3–15% of the total PM_2.5_ mass [6].

Oxidative stress and inflammation are considered important mechanisms of action for particle-generated cardiovascular diseases and cancer, with the latter also believed to be partially attributed to polycyclic aromatic hydrocarbons (PAHs) in the DE particle fraction as summarized in a recent review [7]. The review concludes that exposure to particles affects the vasomotor function, fibrinolysis system and heart rate variability (HRV). Particle exposure may affect the arterial blood vessels to increase vasoconstriction and reduce vasodilation [8]. The effect of DE has been investigated in a number of short-term studies (i.e. few hours) where subjects were typically exposed in a chamber under controlled conditions. This has demonstrated reduced responsiveness to vasodilator-induced vasodilation [9–14], whereas HRV measurements have shown null or mixed results [15–18]. Lung function measurements have shown essentially null results [14, 19–25]. Effects on airways inflammation have consistently been observed in chamber studies [21, 23, 24, 26, 27] with mixed results for systemic inflammation [9–11, 16, 18, 23, 25, 28–30]. In contrast to vascular, lung function and inflammation endpoints, only very few controlled DE exposure studies have included endpoints related to genotoxicity. To the best of our knowledge only one study has assessed DNA strand breaks and oxidatively damaged DNA in humans after controlled DE exposure in a chamber study and this showed no effect [31]. The same endpoints, measured by the alkaline comet assay, are standard genotoxicity tests in particle toxicology and molecular epidemiology on air pollution exposures [32].

The present study investigated real-life exposure to DE in diesel-powered trains. The commuter railway system in the greater Copenhagen area, Denmark, consists of both electric and diesel trains. Some of the diesel trains are relatively old (from the 1980s) and have rather high emission rates of PM. In addition, the DE plume from the locomotive penetrates to the interior of the passenger carriages, giving rise to high particle exposure as demonstrated by net increases of PM_2.5_ (36 μg/m^3^), UFP (2 × 10^5^ particles/cm^3^) and black carbon (8.3 μg/m^3^) compared to the concentrations in electric trains [33]. The high particle concentration in diesel-powered trains has caused concerns about possible long-term health effects of train staff and commuters. In order to investigate the effect of real-life DE exposure, 29 volunteers were recruited to participate in scenarios involving staying inside the first passenger carriage of regional trains 6 h/day for 3 consecutive days. Each volunteer was scheduled for two scenarios in diesel trains and one scenario in an electric train. The study design and the duration of the exposure were overall similar to, and inspired by a previous study on particle exposure in firefighters, which demonstrated elevated levels of PAH metabolites in urine, decreased microvascular function and increased levels of DNA damage in peripheral blood mononuclear cells (PBMCs) in conscripts immediately after a 3 day training course in firefighting as compared to 2 weeks before and 2 weeks after [34, 35]. The biomarkers included lung function, microvascular function, HRV and systemic levels of acute phase proteins and cell adhesion molecules, DNA damage in PBMCs and urinary excretion of PAH metabolites.

## Results

### Exposure characterization

**Fig. 1:**
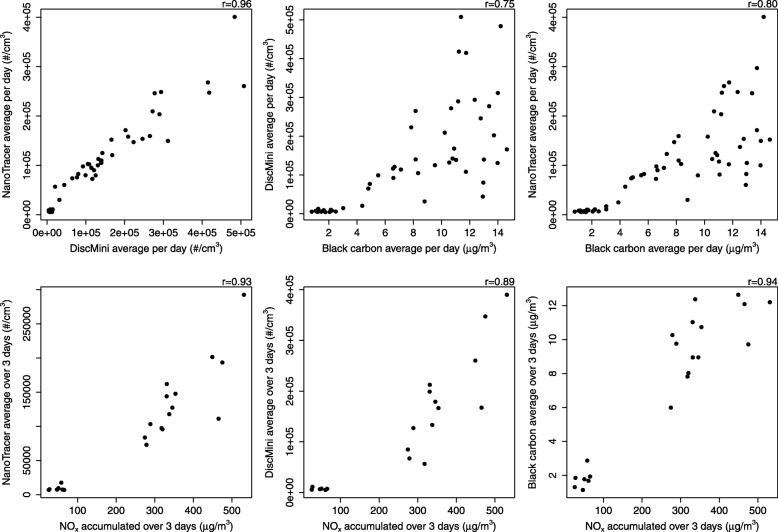
Correlation between air pollution components in diesel and electric trains. The data corresponds to 63 (UFP and black carbon measured with NanoTracer equipment and Aethalometer, respectively) and 55 (UFP measured with DiscMini equipment) days of exposure. Nitrogen oxides were measured over 3 days, corresponding to 18 periods

In total, there were 54 and 29 person scenarios of exposure in diesel and electric trains, respectively. The exposure assessment showed a clear contrast in exposure levels between diesel and electric scenarios for concentrations of black carbon, UFP and nitrogen oxides (Table 1). The average concentrations of UFP were 15 to 23 fold higher in diesel trains, measured with NanoTracer and DiscMini portable devices, respectively. All the air pollution components were highly correlated (Fig. 1). More details on the exposure assessment and results can be found elsewhere [33].

**Table 1:** Black carbon, ultrafine particles and nitrogen oxides concentrations and contrast between diesel and electric trains

Exposure	Electric (*n* = 29)	Diesel (*n* = 54)	Mean difference (95% CI)
Black carbon (μg/m^3^)	1.8 (0.5)	10.3 (2.0)	8.5 (7.9; 9.1)***
Ultrafine particles from DiscMini (#/cm^3^) ^a)^	8100 (2400)	189,200 (91,900)	181,000 (153,700; 208,400)***
Ultrafine particles from NanoTracer (#/cm^3^)	9100 (3500)	133,400 (52,100)	124,300 (110,000; 138,500)***
NOx (μg/m^3^)	45 (16)	363 (73)	317 (297; 338)***
NO_2_ (μg/m^3^)	18 (9)	54 (16)	36 (31; 42)***

The exposure was assigned to study participants (study participants rode the trains in groups of different sizes. The exposure average levels for each calendar day were assigned to all members of the relevant group). Exposure levels in both scenarios are presented as mean and standard deviation. PM_2.5_, polycyclic aromatic hydrocarbons and aldehydes are not assigned to study participants, as the data were not collected throughout all the study period. ^a)^ missing values for DiscMini equipment indexed to four study persons for the exposure scenarios (*n* = 46 diesel and *n* = 25 for electric). ***p < 0.001

### Association between train scenarios and biomarkers

**Fig. 2:**
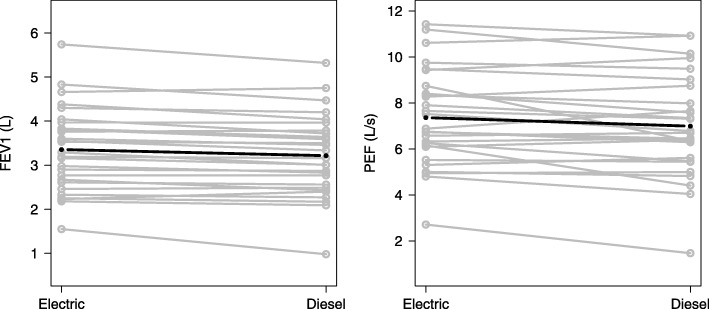
Lung function parameters after exposure in electric and diesel trains. The circles in grey represent individual lung function parameters for electric train (one exposure scenario) or diesel train (mean of two exposure scenarios). The dark line represents the mixed-effects model without adjustments. Lower levels of FEV1 and PEF were observed on group level (solid symbols) after exposure in diesel trains (*p* < 0.05)

Table 2 presents the estimated effect of diesel exposure on the assessed biomarkers as compared to electric trains. The lung function measurements included forced vital capacity (FVC), forced expiratory volume after 1 s (FEV1) and peak expiratory flow (PEF). FEV1 and PEF showed a small but significant decrease after exposure to DE. Figure 2 shows the individual results for the lung function measurements; the mean biomarker level of two measurements is shown.

**Table 2:** Outcome levels for electric and diesel scenarios and percent changes in biomarker levels

Biomarker		Electric (mean ± SD)	Diesel (mean ± SD)	% Change (95% CI)	*p*-value
Urinary excretion	1-OHP (μmol/mol creatinine)	0.049 ± 0.083	0.027 ± 0.019	−12.2 (− 32.3; 14.0)	0.331
	2-OHF (μmol/mol creatinine)	0.075 ± 0.067	0.070 ± 0.061	0.6 (− 17.3; 22.4)	0.952
	1-NAPH (μmol/mol creatinine)	0.851 ± 0.603	0.671 ± 0.560	−20.0 (−44.3; 4.3)	0.107
	2-NAPH (μmol/mol creatinine)	2.084 ± 2.272	1.861 ± 1.679	−0.4 (−20.7; 25.2)	0.975
Lung function	FVC (L)	4.20 ± 1.24	4.18 ± 1.16	−2.3 (−4.7; 0.25)	0.077
	FEV1 (L)	3.32 ± 0.96	3.24 ± 0.96	−3.6 (−5.5; −1.6)	0.0003***
	FEV1/FVC (%)	79.1 ± 6.8	77.2 ± 9.2	−1.8 (− 3.8; 0.2)	0.073
	PEF (L/s)	7.26 ± 2.13	7.15 ± 2.42	−5.6 (− 10.7; − 0.5)	0.031*
Cardiovascular function	Ln.RHI	0.57 ± 0.25	0.55 ± 0.28	−1.4 (− 26.5; 23.7)	0.913
	NIV	2.26 ± 1.06	2.21 ± 0.71	− 3.7 (− 34.6; 27.3)	0.817
	pNN50 (%)	0.06 ± 0.08	6.54 ± 0.07	4.6 (− 13.8; 23.0)	0.626
	RMSSD (ms)	41.84 ± 30.93	36.39 ± 19.02	− 3.5 (− 22.1; 19.7)	0.326
	SDNN (ms)	48.16 ± 24.29	44.24 ± 16.76	−2.1 (− 14.9; 12.7)	0.773
	LF (ms^2^)	160.2 ± 59.5	195.9 ± 78.8	16.5 (5.9; 27.0)	0.002**
	HF (ms^2^)	153.6 ± 57.6	151.8 ± 67.5	2.0 (− 7.5; 11.5)	0.681
	LF/HF	1.27 ± 0.83	1.70 ± 1.37	18.5 (−5.5; 48.6)	0.141
	AI normalized to heart rate of 75 bpm (%)	−8.26 ± 11.79	−6.26 ± 14.68	1.6 (−2.2; 5.5)	0.405
	DP (mm Hg)	81.2 ± 11.4	82.3 ± 10.8	1.2 (−2.9; 5.2)	0.566
	SP (mm Hg)	135.1 ± 16.1	134.4 ± 17.9	−1.0 (−4.9; 2.9)	0.630
DNA damage	SB (lesions/10^6^ bp)	0.12 ± 0.13	0.18 ± 0.13	46.3 (5.0; 100.9)	0.025*
	Fpg-sensitive sites (lesions/10^6^ bp)	0.62 ± 0.15	0.58 ± 0.12	−5.0 (−11.1; 1.1)	0.109
Adhesion molecules	ICAM-1 (ng/mL)	35.04 ± 7.28	34.34 ± 6.88	−2.5 (−8.3; 3.7)	0.426
	VCAM-1 (ng/mL)	134.2 ± 36.2	129.6 ± 35.8	−3.2 (−10.5; 4.7)	0.416
Acute phase proteins	SAA (mg/L)	32.09 ± 41.51	36.46 ± 47.35	11.1 (−17.8; 50.2)	0.493
	CRP (mg/L)	1.83 ± 2.29	1.90 ± 2.48	−12.3 (−47.5; 46.4)	0.615

Percent change was estimated by mixed-effects model adjusted for age and sex, comparing diesel with electric scenarios

*CI* confidence interval, *SD* standard deviation, *1-OHP* 1-hydroxypyrene, *2-OHF* 2-hydroxyfluorene, *1-NAPH* 1-naphthol, *2-NAPH* 2-naphthol, *FVC* forced vital capacity, *FEV1* forced expiratory volume in one second, *PEF* peak expiratory flow rate, *Ln.RHI* reactive hyperemia index with natural logarithmic transformation (the percent change was back transformed), *NIV* nitroglycerin-induced vasodilation, *pNN50* proportion of successive NN intervals differing by more than 50 milliseconds divided by total number of NN intervals, *RMSSD* square root of the mean squared differences of successive NN intervals, *SDNN* standard deviation of all NN intervals, *LF* low frequency component (0.04–0.15 Hz), *HF* high frequency component (0.15–0.4 Hz), *AI* augmentation index, *DP* diastolic blood pressure, *SP* systolic blood pressure, *SB* DNA strand breaks, *ICAM-1* intercellular cell adhesion molecule-1, *VCAM-1* vascular cell adhesion molecule-1, *SAA* serum amyloid A, *CRP* C-reactive protein; *p < 0.05; **p < 0.01; ***p < 0.001

**Fig. 3:**
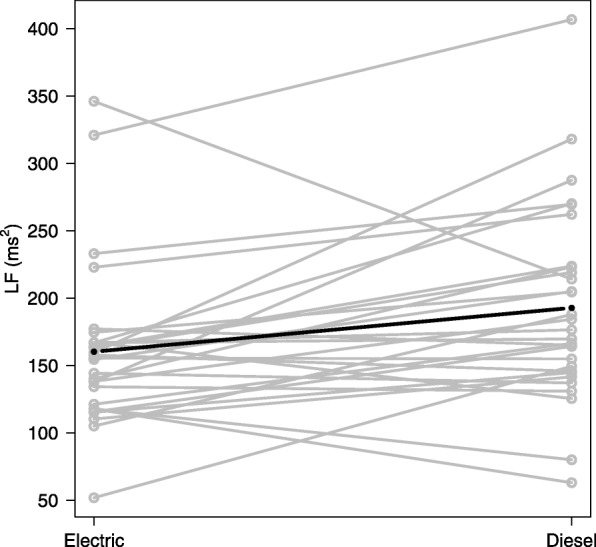
Low frequency (LF) component of heart rate variability measured after exposure in electric and diesel trains. The circles in grey represent LF measurements in electric train (one exposure scenario) or diesel train (mean of two exposure scenarios). The dark line represents the mixed-effects model without adjustments. Higher LF levels were observed on group level (solid symbols) after exposure in diesel trains (*p* < 0.05)

The microvascular function was measured by reactive hyperemia, using EndoPAT, and reported as the logarithmically transformed data (Ln.RHI). Neither this, nor nitroglycerin-induced vasodilation differed between the exposure scenarios, indicating a lack of detectable effect on endothelial-dependent and endothelial-independent vasomotor responses. As expected the nitroglycerin-induced vasodilation was larger (2.2) than the reactive hyperemia-mediated vasodilation (i.e. 1.7, back-transformed from Ln.RHI = 0.57). The only significant change in HRV measurements was an increase in the low frequency-domain component (LF) (Fig. 3), whereas time-domain components were unaffected.

**Fig. 4:**
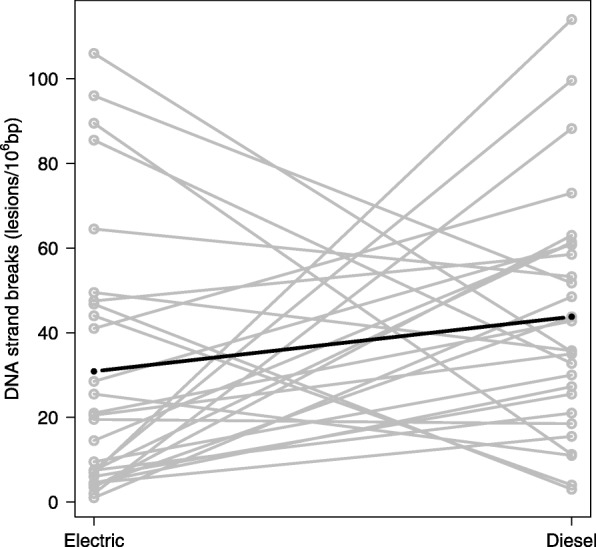
DNA strand breaks in peripheral blood mononuclear cells after exposure in electric and diesel trains. The circles in grey represent DNA strand breaks in electric train (one exposure scenario) or diesel train (mean of two exposure scenarios). The dark line represents the mixed-effects model without adjustments. Higher levels of DNA strand breaks were observed on group level (solid symbols) after exposure in diesel trains (*p* < 0.05)

DE exposure was associated with increased level of DNA strand breaks in PBMCs (Fig. 4), whereas the level of formamidopyrimidine DNA glycosylase (Fpg)- sensitive sites was unaffected by exposure. Markers of systemic acute phase proteins and soluble adhesion molecules were unaffected by exposure.

There was no exposure-related effect on urinary excretion of PAH metabolites used as biomarkers of exposure. The background levels (from electric scenario) of the creatinine adjusted PAHs showed large variation across individual subjects, from below the limit of quantification to 0.377 μmol/mol creatinine for 1-hydroxypyrene (1-OHP), 0.012 to 0.278 μmol/mol creatinine for 2-hydroxyfluorene (2-OHF), 0.117 to 2.55 μmol/mol creatinine for 1-naphthol (1-NAPH) and 0.283 to 10.6 μmol/mol creatinine for 2-naphthol (2-NAPH), respectively.

### Association between air pollution components and biomarker levels

In the second step of the analysis, biomarkers that were statistically significant or with borderline statistical significance (defined as 0.05 > *P* < 0.10) different for the two exposures scenarios were subsequently included in tests for association between individual air pollution components and biomarkers. Table 3 shows the direction of associations between exposure levels (UFP, BC and nitrogen oxides) and effects on biomarkers that showed statistical or borderline statistical significance in the overall test for effect for diesel versus electric trains. Overall, the measurements of lung function were inversely associated with levels of particles and nitrogen oxide levels. Likewise, LF and DNA strand breaks were positively associated with both particles and nitrogen oxides.

**Table 3:**
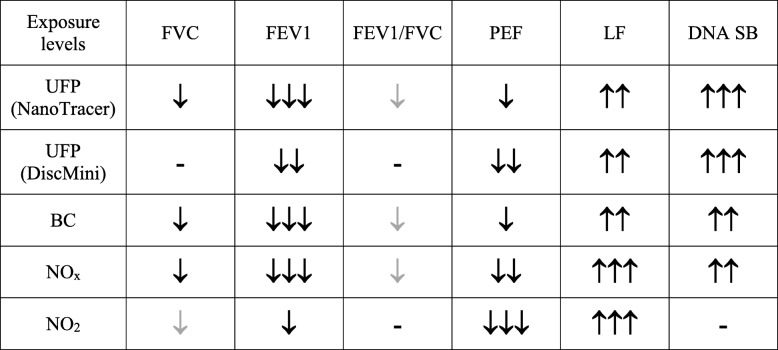
Association between exposure levels and biomarkers estimated by mixed-effects model adjusted for age and sex

Exposure levels are averages of 3 days

*UFP* ultrafine particles, *BC* black carbon, *FVC* forced vital capacity, *FEV1* forced expiratory volume in one second, *PEF* peak expiratory flow rate, *LF* low frequency component, *DNA SB* DNA strand breaks

, , ,  *p* ≤ 0.08; < 0.05; < 0.01; < 0.001, respectively

Table 4 presents the association between each of the 3 days exposure levels from direct-reading instruments and effects assessed on biomarkers sampled on day 3, at the end of the exposure scenario, estimated for the biomarkers that showed statistically or borderline statistical significance in the overall test for effect for diesel versus electric trains. There were no consistent trends between exposure (UFP and BC) and outcomes.

**Table 4:** Estimates for associations between daily averages of exposure levels and biomarkers (91% CI)

Exposure markers	FVC (L)	FEV1 (L)	FEV1/FVC	PEF (L/s)	LF (ms^2^)	DNA SB (lesions/10^6^ bp)^a^
UFP (NanoTracer) (#/cm^3^)						
Day 1	−0.7E−06 (−1.4E−06; −0.04E-06)*	-0.7E-06 (− 1.2E-06; − 0.2E-06)**	-0.6E-05 (− 1.7E-05; 0.5E-05)	−2.2E− 06 (−4.3E− 06; − 0.08E− 06)*	2.2E-04 (0.5E-04; 3.9E-04)*	4.1E-06 (1.3E-06; 6.9E-06)**
Day 2	-0.6E-06 (− 1.1E-06; − 0.02E-06)*	-0.6E-06 (−0.9E-06; − 0.2E-06)**	-0.8E-05 (−1.7E-05; 0.04E-05)	− 1.1E− 06 (− 2.7E− 06; 0.6E-06)	1.3E-04 (0.03E-04; 2.6E-04)*	4.9E-06 (2.8E-06; 7.0E-06)***
Day 3, sampling day	-0.7E-06 (− 1.4E-06; 0.05E-06)	-0.8E-06 (− 1.3E-06; − 0.3E-06)**	−1.0E-05 (− 2.2E-05; 0.1E-05)	−3.1E− 06 (−5.2E-06; − 0.9E-06)**	2.8E-04 (1.2E-04; 4.3E-04)***	3.9E-06 (0.7E-06; 6.9E-06)*
UFP (DiscMini) (#/cm^3^)						
Day 1	-0.6E-06 (− 1.2E-06; 0.01E-06)	−5.2E− 07 (− 9.2E-07;- 1.2E-07)*	-0.5E-05 (− 1.5E-05; 0.5E-05)	−1.9E− 06 (− 3.7E-06; − 0.1E-06)*	1.7E-04 (0.3E-04; 2.9E-04)*	3.9E-06 (1.6E-06; 6.1E-06)***
Day 2	-0.4E-06 (−0.8E-06; 0.06E-06)	−3.2E− 07 (− 5.5E-07; − 0.9E-07)**	-0.4E-05 (− 0.9E-05; 0.2E-05)	−1.2E− 06 (− 2.6E-06; 0.1E-06)	1.4E-04 (0.5E-04; 2.3E-04)**	2.7E-06 (1.0E-06; 4.4E-06)**
Day 3, sampling day	-0.5E-06 (− 1.0E-06; 0.02E-06)	−5.2E− 07 (− 8.6E-07; − 1.8E-07)**	-0.8E-05 (− 1.6E-05; 0.06E-05)	− 2.3E-06 (− 3.8E-06; − 0.8E-06)**	1.5E-04 (0.4E-04; 2.5E-04)**	2.6E-06 (0.6E-06; 4.7E-06)*
BC (μg/m^3^)						
Day 1	−1.5E-02 (−2.8E-02; − 0.3E-02)*	−1.4E-02 (− 2.2E-02; − 0.5E-02)**	−1.2E-01 (− 3.3E-01; 0.9E-01)	−5.4E-02 (− 9.1E-02; − 1.7E-02)**	4.5 (1.8; 7.2)**	4.3E-02 (− 1.2E-02; 9.8E-02)
Day 2	−1.2E-02 (− 2.2E-02; − 0.1E-02)*	−1.3E-02 (− 1.9E-02; − 0.5E-02)***	−1.6E-01 (− 3.3E-01; 0.1E-01)	−3.1E− 02 (− 6.3E-02; 0.2E-02)	3.1 (0.7; 5.6)*	6.6E-02 (2.0E-02; 11.2E-02)**
Day 3, sampling day	-0.8E-02 (− 1.8E-02; 0.2E-02)	−1.1E-02 (− 1.8E-02; − 0.4E-02)**	−1.8E-01 (− 3.4E-01; − 0.2E-01)*	−3.0E-02 (− 6.0E-02; 0.06E-02)	3.4 (1.3; 5.5)**	6.6E-02 (2.3E-02; 10.9E-02)**

Estimated by mixed-effects model adjusted for age and sex. The values in the table are the beta-estimates of the linear associations (linear mixed model for each day and pollutant)

UFP, ultrafine particles; BC, black carbon; FVC, forced vital capacity; FEV1, forced expiratory volume in one second; PEF, peak expiratory flow rate; LF, low frequency component; DNA SB, DNA strand breaks. The exposure data were collected during 3 days and biomarkers data assessed from sampling in the 3rd day of exposure. Day 1 corresponds to the first day in the trains, two days before sampling; day 2 corresponds to the second day in the trains, one day before sampling; and day 3 to the third day in the trains and the sampling day

^a^SB was transformed with cubic root

*,**,***, *p* < 0.05; < 0.01; < 0.001, respectively

## Discussion

This study shows that DE exposure in carriages of diesel-powered trains, for 6 h per day for three consecutive days, was sufficient to cause a reduction of lung function and systemic effects in terms of altered heart rate variability and increased levels of DNA strand breaks in PBMCs in healthy volunteers.

The reduction in lung function after DE exposure although small was remarkably consistent between the subjects. Nevertheless, the study participants could not be blinded for the exposure, which could potentially influence their effort in the spirometry performance. This is an unavoidable study limitation. We minimized this potential bias through the study design, which included a repetition of the diesel scenario, and 21-day wash out periods. It was not possible to separate the acute and late effects of DE exposure in the present study where exposure was repeated for three consecutive days before measurement of biomarkers. Similar associations between lung function measures and NO_X_ gases and particles were found, indicating that effects of specific DE constituents cannot be differentiated in the current study. A study showed that DE exposure (300 μg/m^3^ for 2 h) had no immediate effect on the lung function, whereas there was a stronger decline in ozone-mediated (300 ppb) FEV1 in the DE exposed group as compared to filtered air the day after the exposure [20]. Other studies on short-term exposure to DE have not demonstrated effects on FEV1 in humans [14, 19–25], whereas one study showed reduced PEF starting at 75 min after the exposure [25]. PEF is reduced as a result of obstruction in the airways, which is seen in asthma patients. In addition to a reduced PEF in our study, there were reduced FEV1 and FVC and a near-normal FEV1/FVC ratio. Reduction of both FEV1 and FVC, and unaltered FEV1/FVC ratio is typically observed in restrictive lung diseases, including fibrosis. However, the current exposure is too short for inducing fibrosis and it is more likely that the DE exposure affected the lung compliance towards decreased ability to expand. This effect could be reversible, although recurrent exposures may have a long-term effect on the lung function.

In the present study, there was unaltered vasomotor function and no effect on systemic levels of acute phase proteins (CRP and SAA). Low-grade systemic inflammation is an integrated part of the suggested mechanism of action of particle-generated toxicity in secondary tissues or cells [7]. It can be speculated that the exposure levels in the current study may not be sufficiently high to induce an acute phase response. There was no effect of DE exposure on vasomotor function in the current study. Other short-term controlled exposures to DE have yielded conflicting results in vasomotor function; blunted vasodilator-induced forearm blood flow in certain studies [9–11] and skin microvascular dysfunction [13]. Studies using flow-mediated vasodilation or reactive hyperemia by EndoPAT have shown unaltered vasomotor function [18, 36, 37]. Interestingly, certain studies have also reported increased vasoconstriction in DE exposed humans [18, 37, 38]. The available results may indicate a decreased sensitivity of vasodilator-induced forearm blood flow, although it should also be noted that the diesel fuels and engine types differ between studies. Our previous studies have indicated that exposure to PM from combustion processes were associated with microvascular dysfunction in elderly subjects [39–43], whereas no effects have been observed in young subjects [44, 45]. The wide age span in the present study might have diluted the effect on vasodilation due to inclusion of young subjects who may not be as susceptible to DE-induced vasomotor dysfunction. However, it should also be noted that a meta-analysis from controlled exposures in animal models indicates that DE (or DEP) exposure produces less effect on vasomotor responses as compared to outdoor air pollution particles and nanoparticles [46]. In contrast to the unaltered vasomotor function, we observed effects in the frequency-domain of HRV (16.5% increase in LF and a statistically non-significant 18.5% increase in LF/HF ratio) that suggests a minor increase in sympathetic to vagal activity [47]. This is consistent with our previous finding of increased LFn after 5 –h exposure to street air in elderly slightly overweight subjects [43]. In contrast, a study with controlled DE exposure at 206 μg/m^3^ for 2 h showed a transient alteration in frequency domain outcomes in terms of increased HF and decreased LF/HF ratio at 3 h post exposure [17]). Two other studies have found unaltered HRV in subjects after short-term controlled DE exposure [15, 16].

We observed increased levels of DNA strand breaks after exposure to DE, whereas there were unaltered levels of DNA oxidation lesions in the same PBMCs. In a previous study on controlled DE exposure (276 μg/m^3^ for 3 h) we observed no effect on these endpoints immediately after exposure [31]. However, a controlled exposure study on traffic-generated air pollution showed a positive association between personal particle number concentration of especially size modes 23 and 57 nm, with high deposition fractions, and levels of Fpg-sensitive sites in PBMCs [48]. Personal exposure to UFP, obtained by bicycling in streets with heavy traffic, was positively associated with levels of Fpg-sensitive sites in PBMCs, whereas there was no effect on levels of DNA strand breaks [49]. On the other hand, elderly and overweight subjects had unaltered levels of DNA strand breaks and Fpg-sensitive sites after exposure to urban street air in a controlled exposure study, whereas the total number of DNA lesions was positively associated with the particle number concentration [50]. Studies on high-dose exposure in animals have demonstrated mixed results for the association between pulmonary exposure to DEP and DNA strand breaks in lung tissue with certain studies showing increased levels of DNA damage [51–53] and no effect [54–56]. The literature on oxidatively damaged guanine lesions is partly flawed by studies that have used non-optimal techniques for measurements of 8-oxodG due to unspecific detection or spurious oxidation of DNA during the processing of samples [57]. Two studies on OGG1- or Fpg-sensitive sites, measured by the comet assay have shown unaltered levels of oxidatively damaged DNA in lung tissue at 24 h after i.t. instillation [55, 56]. A study on repeated inhalation (20 mg/m^3^ for 1.5 h on 4 consecutive days) showed unaltered levels of Fpg-sensitive sites and 8-oxodG in lung tissue of wild-type mice [54]. Interestingly, a previous study had shown that a single inhalation exposure to 80 mg/m^3^ of DEP increased the levels of 8-oxodG in mice, whereas the same total administered dose on four consecutive days increased the expression of OGG1 accompanied by unaltered levels of 8-oxodG [58]. This indicated that a high bolus exposure may saturate the DNA repair system, whereas the oxidative damage to DNA can be efficiently removed when DEP is administered in multiple lower doses.

The contrast between exposure levels in passenger carriages of diesel driven trains and electric trains in the present study is clear, as indicated by the net average difference of 8.5 μg/m^3^ in BC (6-fold), 1.2 × 10^5^–1.8 × 10^5^ particles/cm^3^ (15–24 fold) and 36 μg/m^3^ NO_2_ (3-fold) during the 6 h exposure periods. In addition, the average difference in PM_2.5_ concentration between diesel and electric trains was 36 μg/m^3^ [33]. Previous studies on controlled DE exposure have used PM concentrations up to 300 μg/m^3^ for 1–3 h in chambers [19–21, 23, 25]. The time-integrated exposure in the present study (216 μg*h/m^3^ per day or 648 μg*h/m^3^ per 3-day exposure period) is similar to previous studies (300–900 μg*h/m^3^). We have previously reported a contrast in the levels of particulate PAHs, but not for gaseous PAHs in the present train exposure scenarios, although the limited number of samples available did not allow a statistical analysis [33]. We observed unaltered urinary excretion of PAH metabolites; however, it is unclear if the current exposure contrast was large enough not to be masked by dietary exposure, despite the precautions made. Moreover, the sampling time (morning of the third day) may have not been optimal to detect urinary PAH metabolites. In a controlled wood smoke exposure study, urinary excretion of nine hydroxylated PAHs reached maximal concentrations within 2.3–19.3 h and returned to background levels 24 h after exposure [59]. 1-OHP has been used in epidemiological studies, showing positive association with air pollution exposure, although confounding from smoking, occupational PAH exposure and environmental tobacco smoke cannot be ruled out [60]. In contrast, stricter control of other PAH exposures from diet and exclusion of smokers in controlled trials ameliorate confounding.

## Conclusions

The present study showed a consistent reduction in lung function and increased levels of DNA strand breaks after exposure to DE inside passenger carriages of diesel-powered trains, whereas the exposure did not affect the level of oxidatively damaged DNA in PBMCs. The only effect on cardiovascular endpoints was an increased LF in the frequency-domain HRV, suggesting an increase in sympathetic to vagal activity. In agreement with other studies on DE exposure, PAH metabolites were not increased in urine. This may be due to lack of contrast in exposure and lack of sensitivity from the biomarker to detect minor increases in PAH exposure from the background levels of PAH exposure from e.g. diet. Overall, the 3-day exposure to DE in diesel-powered trains was associated with lung and systemic effects.

## Methods

### Study participants

The participants were recruited by registering the study on a human trial web platform (forsoegsperson.dk) and through flyers handed out in the area of Copenhagen, Denmark. We enrolled 33 self-reported healthy, non-asthmatic, without prescribed medication, non-smoking (defined as cessation of smoking at least 1 year before enrollment) and non-pregnant participants living in the Copenhagen region. One subject was excluded after further medical examination and three dropped out before completion. From the 29 participants that completed the study, 4 only participated in two exposure scenarios, failing the participation in the third, for personal reasons. They were included in the analysis since they completed the contrast scenarios. Table 5 presents the general characteristics of the study participants. The age ranged from 21 to 71 years. Fifteen participants had a body mass index (BMI) between 18.6 and 24.8 Kg/m^2^, 10 between 25.2 and 28.1 Kg/m^2^ and four between 30.8 and 39.0 Kg/m^2^.

**Table 5:** Characteristics of the study participants at the control measurements (mean (±SD))

Characteristics	Females (*n* = 15)	Males (*n* = 14)	Total (*n* = 29)
Age (years)	34.7 (±14.7)	43.4 (±17.6)	38.9 (±16.5)
Height (cm)	166.6 (±4.7)	181.4 (±10.1)	173.7 (±10.7)
Weight (kg)	71.5 (±17.5)	81.4 (±16.5)	76.3 (±17.4)
BMI (kg/m^2^)	25.6 (±5.3)	24.7 (±3.8)	25.2 (±4.6)
FVC (%)	92.7 (±15.6)	91.2 (±21.5)	92.0 (±18.2)
FEV1 (%)	89.4 (±16.6)	89.4 (±19.2)	89.4 (±17.5)
FEV1/FVC (%)	96.3 (±7.6)	98.0 (±6.5)	97.1 (±7.0)
PEF (%)	87.3 (±20.4)	85.4 (±18.2)	86.4 (±19.1)

*SD* standard deviation, *BMI* body mass index, *FVC* forced vital capacity, *FEV1* forced expiratory volume in one second, *PEF* peak expiratory flow rate. The lung function parameters are presented as the percentage of the predicted value from the general population in the NHANES III survey

### Study design

The study participants travelled 6-h per day during three consecutive days (always on Tuesday, Wednesday and Thursday) inside diesel or electric trains running in the Zealand region, exposure scenarios that have been previously described [33]. The study design was a crossover, repeated measures, where participants served as their own control with a randomized order of exposure inside diesel or electric trains. The diesel scenario exposure was repeated twice, to account for the observed exposure levels and daily variations (four participants only participated in one diesel train exposure). All exposure scenarios were separated by 2-week periods. The participants were instructed to wear a mask (3 M Aura™ 9320+, USA) on the way from home to the train station and back home on the exposure days, to prevent other ambient air PM exposure in their transportation activity. At the end of the third day in each scenario, the participants walked for 15 min from the station to the university facility where the biological samples were collected. They did not use the mask on this walking trip, with the instruments collecting data on exposure. They were also told to avoid consumption of smoked food on the exposure days, and were offered a packed lunch consisting of a sandwich (of humus, tuna or chicken with salad and sauce), fruit, water and a muesli bar every day on the trains. In the morning of the third day in each exposure scenario, the participants gave a urine spot sample, and in the end of the day, we sampled blood and measured lung and cardiovascular function. The participants filled four questionnaires, one for housing characteristics and lifestyle and one for each sampling day about food, activity and medication intake. The volunteers were travelling in groups of 3 to 6 subjects and the entire study was completed within a 7-month period, from May to end of November 2017, with intermission during July.

### Exposure assessment

The study participants carried instruments to monitor UFP (DiscMini and NanoTracer), BC (MicroAeth AE51) and nitrogen oxides (passive samplers Ogawa) as described previously [33]. Daily averages of UFP and BC were determined (without being synchronized and including the data collected when the study participants walked from the station to the university facility where the biological sampling was performed) and averages over the 3 days of each exposure scenario were allocated to each study participant. For nitrogen oxides the accumulated averages exposure were used and also allocated to each study participant. Two measurement days with DiscMini were excluded due to battery failure. A more comprehensive description of exposure data has been published [33], although with a different treatment, as here all the collected data was included in the daily averages, without synchronization start and end times and without eliminating days with delays in the trains.

### Lung function

Lung function was assessed with the EasyOne™ Sprirometer 2001 (ndd, Medical technologies, Zurich, Switzerland), in diagnostic mode, measuring FVC, FEV1 and PEF. All measurements were performed after careful instructions and with the participants standing. At least three acceptable manoeuvres were performed to obtain reproducible tracings. The measurements were performed 30–60 min after ending the exposure scenario. Two spirometer results were eliminated due to deficient test quality.

### Urine and blood sampling and analysis

First morning urine samples were delivered by the participants in 120 mL flasks, aliquoted and stored at − 20 °C. Peripheral venous blood samples were collected at the Department of Public Health laboratory facilities in Vacutainer cell preparation tubes (CPT™, Becton Dickinson A/S, Brøndby, Denmark) for isolation of PBMCs and ethylenediaminetetraacetic acid (EDTA)-coated tubes for plasma preparation. The samples were stored at − 80 °C in preserving medium, as previously described [34]. It was not possible to collect (and further analyse) 3 samples of blood.

#### Analysis of biomarkers of exposure in urine

Levels of 1-OHP, 2-OHF, 1-NAPH and 2-NAPH in urine were measured after solid phase extraction (SPE) using liquid chromatography with tandem mass spectrometry (LC-MS-MS). In brief, 1 ml of urine was mixed with a buffer solution and deconjugated (β-glucoronidase, 37 °C, 18–20 h), after which deuterium-labeled internal standards were added. The samples were loaded onto pre-washed 500 mg C18 SPE-columns (Bond Elut, Agilent Technologies) and subsequently washed using 6 ml methanol:water (1:3) and 6 ml water. The SPE-columns were dried overnight at 55 °C and eluted with 3 ml methanol. The extract were evaporated to dryness under a gentle stream of nitrogen and reconstituted in 300 μL methanol. The extracts were analyzed on an Agilent LC-MS-MS (series 6460) using a Phenomenex C18, 100 Å, 100 × 2 mm column with a gradient of water and methanol. 1-OHP and 2-OHF were quantified using d_9_–1-OHP while d_8_–2-NAPH was used for 1-NAPH and 2-NAPH. The limit of quantification was set to 10 times the signal-to-noise ratio.

All urine concentrations were standardized for diuresis with the concentration of creatinine as previously described [61].

#### Analysis of biomarkers of effect in blood

The concentrations of SAA and CRP in plasma were determined by the enzyme-linked immunosorbant assay kits from Invitrogen (CA, USA) and IBL International GMBH (Hamburg, Germany), as previously described [62]. Plasma levels of soluble ICAM-1 and VCAM-1 were measured with BD cytometric bead array system, using Accuri CFlow Plus software (BD Bioscience) as described previously [63]. DNA damage was assessed by levels of DNA strand breaks and Fpg-sensitive sites using the comet assay as described elsewhere [64]. The number of Fpg-sensitive sites was obtained as the difference in scores of parallel slides incubated with and without Fpg (gift from Professor Andrew Collins, University of Oslo, Norway). These scores were transformed to lesions per 10^6^ base pairs (bp) by means of a calibration curve based on induction of DNA strand breaks by ionizing radiation (0–2.5 Gy), which has a known yield. We used an investigator-specific conversion factor of 0.0162 lesions/10^6^ bp per score in 0–100 range, based on the assumption that an average molecular weight of a DNA bp is 650 Da and one Gy yields 0.29 breaks per 10^9^ Da DNA [65]. Assay control (i.e. cryopreserved samples of THP-1 cells exposed to 5 mM KBrO_3_ for 1 h at 37 °C as recommended elsewhere [66]. The assay controls were 1.29 ± 0.12 and 0.14 ± 0.05 for Fpg-sensitive sites and DNA strand breaks, respectively (mean ± SD, *n* = 10).

### Cardiovascular function

RHI, HRV and augmentation index were measured non-invasively using the portable EndoPAT2000 (Itamar Medical Ltd., Israel), as previously described [35]. The cardiovascular function was the last measurement performed on the study participants (1–2 h after end of exposure). Blood pressure was measured with a single measurement using an automatic upper arm blood pressure monitor (Microlife Colson BP 3BXO-A, Widnau, Switzerland), before the peripheral arterial tonometry (PAT) measurement and in the contralateral arm (control arm), where the blood sample was also taken. Pneumatic sensors were placed on the index fingers to measure pulse volume changes in three test phases: a baseline recording (6-min), a brachial arterial occlusion of one of the arms (5-min), and a post-occlusion recording of the induced reactive hyperemia response (5-min), with reference to the finger probe on the control arm. Additionally, we also measured the vasodilation induced in the control arm after sublingual administration of 0.25 mg of nitroglycerin. The nitroglycerin-induced vasodilation was calculated as the ratio of one-minute average amplitudes of the PAT signal after and before administration, chosen from the 5 min signal at baseline after reactive hyperemia effect and the peak reached during the 15 min after nitroglycerin treatment. The nitroglycerin was administrated only to 10 participants in both exposure scenarios because of limited medical supervision or a possible history of migraine precluded the administration of nitroglycerin. The EndoPAT software determines the HRV based on 5 min from the baseline recording, including time domain (SDNN, pNN50 and RMSSD), high (HF) and low frequency (LF) components as well as the ratio LF/HF. It is also from the baseline recording that the augmentation index is determined. All the measurements were done with the participants resting seated, in a quiet room. HRV and LnRHI measurements had 6 missing values.

### Statistics

The results were analysed in a hierarchical approach: first, the effect of diesel exposure as compared to electric trains on all the assessed biomarkers was assessed, and secondly, for biomarkers with statistical significance or border line significance, the associations between air pollution components and biomarkers were assessed. We analysed our results in R statistical environment by linear mixed-effects model using the package *lme4* [67]. As fixed effects we used factorial variables of exposure (diesel/electric) and sex and continuous variable of age. The analyses were adjusted for sex and age because we had missing data. As random effects we used by-participant intercepts. *P*-values were obtained with the function *glht* from *multcomp* package [68]. We tested the interaction of the order of exposure scenario (electric or diesel) for the relevant biomarkers (with significance or border line significance in the first analysis) and there were no significant interactions. The percent changes were determined by dividing the estimate change with the intercept value and multiplying with 100, except for RHI, augmentation index, SDNN, RMSSD, LF/HF, FVC, ICAM-1, VCAM-1, 2-OHF, 2-NAPH, SAA and CRP that were natural logarithmically transformed and DNA strand breaks and 1-OHP that were transformed with cubic root, and therefor percent changes were obtained from (EXP_estimate_-1)*100 and (((((estimate+intercept)^3^-intercept^3^) + intercept)/intercept)-1)*100, respectively. The residuals were checked for normality with Shapiro-Wilk test, kurtosis and graphically with histogram and Q-Q plot. Augmentation index, SDNN, RMSSD, LF/HF, FVC, ICAM-1, VCAM-1, 2-OHF, 2-NAPH, SAA, and CRP, showed better distributions after natural logarithmic transformation and DNA strand breaks and 1-OHP after cubic root transformation. Associations that were statistically significant in mixed effects models have been depicted in graphs with the mean of the two measurements for each study participant in the DE scenario. The corresponding univariate analyses of the data in the graphs are mixed effects models without control for age and sex (similar to paired sample t-test).

## Data Availability

The datasets analysed during the current study are available from the correspondent author on reasonable request.

## Abbreviations

BC: Black carbon
BMI: Body mass index
bp: Base pairs
CRP: C-reactive protein
DE: Diesel exhaust
DEP: Diesel exhaust particles
FEV1: Forced expiratory volume in one second
Fpg: Formamidopyrimidine DNA glycosylase
FVC: Forced vital capacity
HF: High frequency component (0.15–0.4 Hz)
HRV: Heart rate variability
LF: Low frequency component (0.04–0.15 Hz)
PAH: Polycyclic aromatic hydrocarbons
PAT: Peripheral arterial tonometry
PBMCs: Peripheral blood mononuclear cells
PEF: Peak expiratory flow
PM: Particulate matter
pNN50: proportion of successive NN intervals differing by more than 50 milliseconds divided by total number of NN intervals
RHI: Reactive hyperemia index
RMSSD: Root mean square of the successive differences
SAA: Serum amyloid A
SDNN: Standard deviation of all NN intervals
UFP: Ultrafine particles
